# Multi-Modal Medical Image Registration with Full or Partial Data: A Manifold Learning Approach

**DOI:** 10.3390/jimaging5010005

**Published:** 2018-12-30

**Authors:** Fereshteh S. Bashiri, Ahmadreza Baghaie, Reihaneh Rostami, Zeyun Yu, Roshan M. D’Souza

**Affiliations:** 1 Department of Electrical Engineering, University of Wisconsin-Milwaukee, Milwaukee, WI 53211, USA; 2 Department of Computer Science, University of Wisconsin-Milwaukee, Milwaukee, WI 53211, USA; 3 Department of Mechanical Engineering, University of Wisconsin-Milwaukee, Milwaukee, WI 53211, USA

**Keywords:** medical image registration, multi-modality, partially overlapped images, manifold learning

## Abstract

Multi-modal image registration is the primary step in integrating information stored in two or more images, which are captured using multiple imaging modalities. In addition to intensity variations and structural differences between images, they may have partial or full overlap, which adds an extra hurdle to the success of registration process. In this contribution, we propose a multi-modal to mono-modal transformation method that facilitates direct application of well-founded mono-modal registration methods in order to obtain accurate alignment of multi-modal images in both cases, with complete (full) and incomplete (partial) overlap. The proposed transformation facilitates recovering strong scales, rotations, and translations. We explain the method thoroughly and discuss the choice of parameters. For evaluation purposes, the effectiveness of the proposed method is examined and compared with widely used information theory-based techniques using simulated and clinical human brain images with full data. Using RIRE dataset, mean absolute error of 1.37, 1.00, and 1.41 mm are obtained for registering CT images with PD-, T1-, and T2-MRIs, respectively. In the end, we empirically investigate the efficacy of the proposed transformation in registering multi-modal partially overlapped images.

## 1. Introduction

Medical image analysis involves various tasks including image segmentation [1], reconstruction [2], classification [3,4], and tumor detection [5], to name a few. In many such applications, the information we seek can be obtained by comparing or fusing two or more images. Inevitable misalignment between images makes the image registration a fundamental component of many of the problems mentioned earlier. Image registration aims to find the best spatial alignment between two or more images (called *reference* and *sensed* [6]) of the same scene, which have been taken at different times or with same/different devices in a single/multi-view fashion [7,8]. The images involved in image registration may have been captured using a same (*Mono-Modal*) or different (*Multi-Modal*) imaging techniques.

Image registration is an ill-posed inverse problem [9]. It carries a list of challenges, such as intensity variations between the reference and the sensed images, missing/partial data, and the lack of unique similarity measure, to name a few [10,11]. Despite all the efforts in studying the problem of medical image registration, which have been carefully reviewed in [8,9,12,13,14,15,16,17], yet, there is no unique technique that works in all circumstances.

Several criteria have been considered in the literature to categorize existing medical image registration methods [8,9,12,13,14,18,19]. Among those, the most widely used taxonomy is the one developed by Maintz and Viergever in 1998 [14], in which nine basic paradigms, including dimensionality, nature of registration basis, nature of transformation, domain of transformation, interaction, optimization procedure, modalities involved, subject, and object were incorporated. Although it provides a comprehensive perspective on the topic, the image overlap criterion (partial or full) has not been taken into account, and hence, not been studied well in the literature.

Image registration with partial overlap is the situation in which, only a portion of one image is captured in the other image. In such a situation, the assumption that there is correspondence between every region in both images is not valid. Image registration with partial overlap has application in non-medical images (e.g., remote sensing), biological imaging (e.g., Electron Microscopy), and medical scans when the field of view in one image is not big enough to cover the whole object of interest or when shadows and view-dependent artifacts occlude structures visible in the other image. In the case of CT and MRIs, a complete cross-sectional image of an organ is taken in the axial plane. However, they are usually reviewed and compared by radiologists using other anatomical planes (e.g., sagittal). Depending on the relative starting and relative ending position of both image stacks, the amount of overlap between the corresponding image slice from both stacks in the sagittal direction may be partial. Other reasons for partial image registration could include: (a) miscommunication between the physician and technician on which image view is needed; (b) the need to compare a past image to a current image when images are inconsistent in view; (c) the physical attributes of a patient may require breaking down a scan into overlapping volumes; or (d) the case of incidental findings. A scenario for the latter is when a scan is acquired for one reason (e.g., low back pain) and the radiologist identifies abnormalities outside the region of interest [20]. The new finding requires another scan with a focus on a different part of the body, resulting in an overlap with a small portion of the first scan. Thus, comparing the new scan with the original one will require image registration with partial overlap. All in all, with the lack of one-to-one correspondence between two images with partial overlap, it is highly unlikely to obtain an accurate registration with most common registration methods [10].

**Figure 1:**
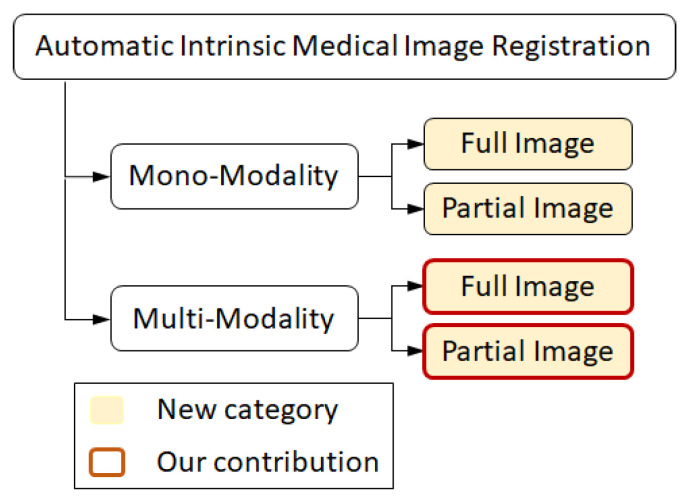
The proposed *complementary taxonomy* of medical image registration methods by considering the imaging modalities and image overlap. We also present a multi-modal image transformation pipeline that assists *multi-modal image registration with full or partial overlap* by its current implementation.

In this research, we present a *complementary taxonomy* concerning the imaging modalities and image overlap. Moreover, we present an image registration pipeline, which provides benefit to multi-modal image registration with both full and partial overlap. Figure 1 highlights our current contribution. Here, we begin with a brief review of recent advances and the computational techniques developed in these categories.

### 1.1. Mono-Modality Medical Image Registration

Mono-modal medical image registration methods aim to register images obtained by the same imaging technique (e.g., CT), and they can be grouped into two sub-categories, including partial and full image registration. The computational task of partial image registration strategy is to align an incomplete or a portion of image data within a complete image; however, in full image registration, the entire region of interest is fully captured in both images. As most effort has been put on image registration with full data, the following reviewed articles fall into the category of registration with full image unless it is noted.

In 1996, Reddy et al. [21] presented an FFT-based technique, which seeks optimal translation, rotation and scale parameters between the reference and sensed images. This extension of phase correlation technique was later known as Fourier–Mellin Transform-based (FMT) image registration method. In 2005, Guo et al. [22] proposed an improved version of FMT in which the conversion from Cartesian to log-polar coordinates was eliminated. The accuracy and robustness of the method with respect to noise was evaluated and verified. Although this method is applicable for images with full and/or partial data, it is constrained to recover scale factor less than 2 and suffers from the poor performance of the cross-power spectral function. In 2011, Pace et al. [23] developed an anisotropic diffusive regularization approach that provides more realistic registration in the presence of sliding motion of organs. The method was validated with the use of both artificial and XCAT software phantom data. Later in 2013, Pace et al. [24] extended their previous work by providing a better optimization method and investigating the accuracy of the proposed method with the use of clinical dataset and lung landmarks. In 2011, Metz et al. [25] addressed the problem of motion estimation for dynamic mono-modal medical images by taking the spatial and temporal smoothness of transformations into account. The software developed based on the proposed method is publicly available to the research community. The application of stochastic optimization strategy in the problem of intensity based mono-modal registration has been also explored, and in 2011, Klein et al. [26] proposed a Robbins-Monro stochastic gradient descent-based algorithm to improve the convergence rate of both rigid and non-rigid registration. In the feature-based group of image registration methods, the accuracy mostly depends on feature matching strategy and control points detection. Inspired by Non-Subsampled Contourlet Transform (NSCT), in 2012, Azzawi et al. [27] implemented a computational method to better detection of salient edges in MRI medical images, and consequently more accurate mono-modal image registration. In 2014, Ghaffari et al. [28] implemented a brand-new similarity measure based on sparse representation in order to deal with non-stationary intensity distortions in mono-modal images. In the group of image registration techniques based on Partial Differential Equations (PDEs), the main challenge is finding a reliable and computationally efficient solver that can handle a large and sparse non-linear system of equations. In 2016, Chumchob et al. [29] introduced a multi-grid algorithm based on local Fourier analysis to solve PDE-based mono-modal image registration problems more efficiently. In 2017, Ghaffari et al. [30] proposed a low-rank matrix-based method to model spatially varying intensity distortion applicable to mono-modal medical image registration and distortion correction.

### 1.2. Multi-Modality Medical Image Registration

In contrast to mono-modal image registration strategies, multi-modal registration algorithms focus on finding correspondence between images generated using various modalities (e.g., CT and MRI), and providing intensive visual information from the fusion of different medical imaging modalities. Comparing to mono-modal registration, multi-modal medical image registration algorithms are more challenging due to the high variability of tissue appearance under different modalities. Despite the challenges, multi-modal image registration is of vital importance to many medical image analysis applications, such as monitoring the progression of diseases (e.g., cancer, aneurysms, etc.), quantifying the effectiveness of treatment mechanisms, surgery planning, and intra-operative navigation, as each imaging modality captures unique tissue features. Similar to the literature review in previous section, the following reviewed articles fall into the category of registration with full data unless it is noted.

In 2006, Periaswamy et al. [10] implemented a general-purpose image/volume registration method based on global and local variations in image intensities. The algorithm was tested on multi-modal images with full data and mono-modal images with partial data; however, it has special shortcomings caused by estimating the deformation field using elastic models. Information theory based metrics, e.g., Mutual Information (MI) [31,32] and its variations, have been widely used for intensity-based inter-modality registration. While MI-based registration is robust, reliable, and fully automated, it suffers from a set of shortcomings such as small convergence rate, low accuracy, and sensitivity to implementation decisions. In 1999, Studholme et al. [33] proposed a Normalized Mutual Information (NMI) metric that can deal with an increase in the value of mutual information due to misalignment when, the sum of the marginal entropy increases faster than the joint entropy. In 2008, Andronache et al. [34] offered an alternative solution to the challenges associated with the estimation of MI between small patches present in the hierarchical subdivision registration strategies. As these global similarity metrics ignore the local structural information, in 2008, Lu et al. [35] tackled the problem of being caught in the local extreme while computing MI. They introduced a novel joint histogram estimation method applicable for rigid and non-rigid multi-modal registration. To revisit the problem of overlap sensitivity of MI-based similarity measures over the course of registration (as discussed in ref. [33]), in 2009, Cahill et al. [36] utilized Cumulative Residual Entropy (CRE) to propose normalized versions of CRE-based NMI and ECC. Authors showed that the proposed similarity measures outperform CRE-based MI similarity measure statistically significant for rigid multi-modal registration. In 2010, Wachinger et al. [37] offered a new approach in registering multi-modal images, assuming that the images obtained from different modalities present similar internal structures. They utilized Laplacian Eigenmap, a manifold learning technique, for structural representation of multi-modal images and evaluated the proposed idea using simulated MRI scans. Later in 2012, Wachinger et al. [38] investigated and compared the performance of entropy and manifold learning-based methods to construct structural representations applicable to medical image registration. In 2012, Heinrich et al. [39] introduced a Modality Independent Neighborhood Descriptor (MIND) that is applicable for registering images across arbitrary modalities. While the descriptor is suitable for different registration methods, it is not rotationally invariant and hence, can not recover strong rotations. Using the descriptor in image registration applications requires the anatomical feature to be present in both modalities. In 2014, Aktar et al. [40] utilized Partial Volume Interpolation (PVI) technique along with the Sum of Conditional Variance (SCV) in developing a volume based multi-modal image registration strategy. The evaluation of the proposed method was limited to registration of simulated MRI modes. In 2015, Oktay et al. [41] presented a Probabilistic Edge Map (PEM) based on a trained structured decision forest that enables structural representation of multi-modal images. The proposed learning-based method relies on human-engineered features and requires a separate training step which, limits its application. In 2016, Simonovsky et al. [42] proposed a deep Convolutional Neural Network (CNN) -based metric for multi-modal image registration and employed it with a continuous optimization framework, which outperformed the widely used MI-based strategy by a meaningful margin in registering T1-T2 MRI images. In 2017, Cao et al. [43] developed a bi-directional image synthesis based technique to solve the problem of CT-MRI pelvic image registration. Most recently, in 2018, Ceranka et al. [44] studied and compared different medical image registration algorithms for the purpose of whole-body MRI mosaicing in a multi-modality fashion, and Li et al. [45] proposed a two-step (coarse and fine) registration method to address issues caused by modality-varied resolution, luminosity, and contrast across retina images.

### 1.3. Motivations and Main Contributions

Each imaging modality has specific advantages and shortcomings. For example, considering CT and MRI as the two widely used imaging modalities that produce detailed, cross-sectional images with comparable spatial resolution, MRI has higher contrast resolution while CT scans are relatively less expensive and fast. Hence, in longitudinal studies, CT has been typically employed as the primary mode of imaging, and MRI has been used for acquiring additional details. In this article, considering the importance of image registration in the accuracy of further analysis and interpretation of medical images, we are motivated to develop a computational algorithm that facilitates multi-modal medical image registration.

Missing and partial data often cause the failure of commonly used multi-modal registration methods. As it is noticed in the literature review, not much effort has been devoted to the problem of registering multi-modal images in the situation where the full image of the object of interest is not available. Lack of effective method in dealing with this problem motivates the next aim of the current article.

Dimensionality reduction algorithms, known as *manifold learning*, have made a big advance in image/data processing domain. They aim to uncover or *learn* a possible low dimensional manifold embedded in a high dimensional space while respecting the intrinsic geometry [46]. These techniques have been widely used in the field of medical image analysis [37,46,47]. However, there are not many structured applications in medical image registration. The capabilities of manifold learning strategies in dealing with high dimensional data motivate the last aim of the current study that is to utilize manifold learning in multi-modal medical image registration.

In the present contribution, we are inspired by the properties of the Laplacian Eigenmap as a manifold learning method and the registration technique presented by Wachinger et al. [37]. Even though the method proposed in [37] provides a smooth convergence to the optimal aligning point of the input images, it fails to obtain a fine-grade registration. Moreover, the method is neither applicable to registering images with partial overlap, nor with deformable registration. Two primary reasons that cause the problem are large patch sizes used to study input images and inefficient manifold alignment technique. Authors lose details of the inner structure of the input images by using patches of size 15×15, which results in vague structural representations (please see Figure 5). When dealing with partial registration with the inner portion of the organ as well as deformable registration, the amount of details that is lost prevents an optimizer from obtaining a precise registration result. It is important to note that using small patches alone cannot help with efficient embedding and accurate registration. The manifold alignment technique and the technical details of implementing the manifold learning play a critical role (please see Figure 8). The list of our main contributions is as follows:

- We improve the technique presented in [37] and implement a multi-modal to mono-modal transformation. The proposed transformation facilitates a direct application of well-founded mono-modal registration techniques on multi-modal medical images as well as recovering strong scales, rotations, and translations. The current contribution falls into the group of multi-modality registration techniques (taxonomy presented in Figure 1). The method is novel in terms of its applicability for both types of images with full and partial overlap.
- Furthermore, we propose a fast and easy-to-use alignment technique that compensates random sign ambiguities caused by reflection of a principal vector. The alignment technique facilitates application of more complex optimization algorithms as well as intensity-based metrics. The proposed intensity transformation addresses the problem of registering multi-modal medical images with partial overlap. We qualitatively examine the performance of the proposed method, where commonly used MI-based multi-modal registration methods fail.
- From the experimental perspective, we present a set of qualitative and quantitative analysis to examine the performance and accuracy of the proposed system in which, a clinical input image was subject to multiple degree of freedom (translation, rotation, and/or scale at the same time). Using multiple datasets, including synthetic and real patients’ human brain images, we obtained a lower mean absolute error across different multi-modality registrations.

The rest of the article is organized as follows. To make the current contribution self-contained, Section 2 discusses the underlying principles and notations. Materials and methods are fully explained in Section 3. The detailed discussion on the implementation of the Laplacian Eigenmap as well as choosing the correct value for the parameters, can be a good reference for researchers. In Section 4 we present a set of experimental validations to examine the quality and quantity attributes of the proposed method. Section 5 further discusses the method and concludes the current contribution.

## 2. Preliminaries

Dimensionality reduction techniques with respect to the transformation that maps high dimensional data into a low dimensional space are categorized into *linear* and *non-linear* methods. Several linear and non-linear techniques have been introduced in all of which a set of eigenvectors associated with the top (or bottom) eigenvalues of a specific matrix are sought for. Therefore, these algorithms are considered as *spectral embedding methods*. The common properties of both types of algorithms are flexibility and simplicity [48].

Dimensionality reduction deals with the following problem: consider a set $x_{1},\ldots ,x_{n}\in M$ of *n* sampled data points on the surface of the manifold M with $x_{i}\in R^{D}$ and we are looking for a set of corresponding points $y_{1},\ldots ,y_{n}$ where $y_{i}\in R^{d}\left(d\ll D\right)$ that preserves specific relevant information of $X=[x_{1},x_{2},\ldots ,x_{n}]$.

In the current contribution, two of the widely used methods, namely Principal Component Analysis (PCA) [49] and Laplacian Eigenmap [50] are mainly employed as linear and non-linear techniques, respectively. Hence, in the current section, we provide the necessary background for the proposed multi-modal to mono-modal transformation by briefly reviewing PCA and Laplacian Eigenmap methods.

### 2.1. Notations

In the current article, boldface upright capital letters and boldface lower-case letters represent matrices and vectors, respectively. Lower-case letters without boldface style represent scalar values and points. Sets are illustrated using capital letters without boldface style.

### 2.2. Principal Component Analysis (PCA)

Two advantages of linear methods, in general, are being easy to compute and providing an explicit mapping from the high dimensional space to the low dimensional embedding [51,52]. The latter property states that new incoming data points can be projected to the same low dimensional space without going through the embedding procedure from the beginning. Among various linear dimensionality reduction algorithms, indeed PCA is the most popular one [53].

PCA aims to find a set of perpendicular directions that data is spread along with (has maximum variance) by considering the relative importance of these directions [54]. PCA is based upon the assumption that the variance of a dataset is a measure of the amount of information stored in the dataset. The higher the variance, the more information stored. Hence, PCA looks for a transformation A that preserves maximum variance [55].

Given the matrix $X\in R^{D\times n}$ whose columns are *D*-dimensional data points, the matrix $X_{c}\in R^{D\times n}$ is defined as the matrix of *centered* data points (the sample mean of each row has been shifted to zero). Then the projections are given by $Y={\mathrm{AX}}_{c}$, in which Y is an (d×n) matrix. PCA optimizes the objective function:

(1) $$\max_{M}\mathrm{var}\left({\mathrm{AX}}_{c}\right)$$

where A is an orthogonal (d×D) matrix. The covariance matrix of Y is:

(2) $$\begin{array}{ccc} \Sigma_{y} & = & E\left[{\mathrm{YY}}^{T}\right]=\frac{1}{n}\left({\mathrm{AX}}_{c}\right){\left({\mathrm{AX}}_{c}\right)}^{T}\\ & = & A\frac{X_{c}X_{c}^{T}}{n}A^{T}={\mathrm{AVA}}^{T} \end{array}$$

where $V=\frac{1}{n}X_{c}X_{c}^{T}=cov\left(X_{c}\right)$ is the covariance matrix of centered data points. From the Equation (2), it is clear that **A** attempts to maximize the cost function $trace({\mathrm{AVA}}^{T})$. By adding a constraint ${∥A∥}^{2}={\mathrm{AA}}^{T}=I$, we make sure that the transformation matrix is orthonormal.

Using *Lagrange multiplier technique*, the maximization problem is simplified to solving the eigenvalue problem of the covariance matrix V:

(3) AV=λA.

According to linear algebra, any matrix in the form of ${\mathrm{MM}}^{T}$ is symmetric. Therefore, **V** is symmetric, which yields to the conclusion that, according to the *spectral theorem*, V is orthogonally diagonalizable and has only *real non-negative eigenvalues*. The orthogonally diagonalizable matrix **V** has *orthogonal non-zero real eigenvectors*.

**Figure 2:**
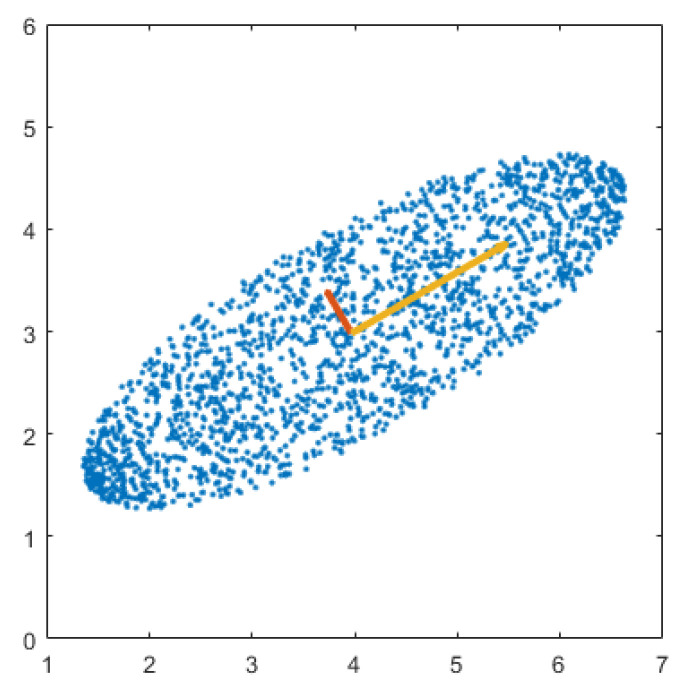
Principal components of a sample synthetic dataset.

It is clear that an (D×D) matrix **V** has maximum number of *D* eigenvalues and *D* eigenvectors of size (D×1). Let $\lambda_{1}\geq \lambda_{2}\geq \ldots \geq \lambda_{D}\geq 0$ (in decreasing order) with corresponding orthonormal eigenvectors $\vec{a_{1}},\vec{a_{2}},\ldots ,\vec{a_{D}}$. The eigenvectors of matrix **V** are *principal components* (or *principal directions*) of data points, along which data points have maximum variance. Principal components of a sample synthetic dataset are illustrated in Figure 2. These principal components make a set of normal basis vectors that projects high dimensional data points into a low dimensional space while preserving most of the information. The desired linear transformation matrix A is made up of the first *d* principal components associated with the first *d* largest eigenvalues [56].

### 2.3. Laplacian Eigenmap

Non-linear dimensionality reduction techniques have drawn great interests due to the incapability of linear methods in revealing nonlinear relationships between data points [48]. In the literature, *non-linear dimensionality reduction techniques* are commonly called *manifold learning*, despite that term technically referring to either linear or nonlinear techniques. Across various manifold learning methods, Laplacian Eigenmap is relatively computationally efficient with well-established computational structure [37]. It is a spectral analysis technique and in close connection to the heat flow [57].

The objective of Laplacian Eigenmap is finding a low dimensional embedding of a high dimensional manifold as a *structural representation*. While preserving local information, it extracts structural information and embeds it in a low dimensional space. The embedded manifold is called structural representation. The new representation benefits from *locality preserving* and *structural equivalence* properties. Locality preserving means the structural representations of two similar patches of a same manifold are similar after being mapped to the new coordinates. On the other hand, structural equivalence is the case when the structural representations of two similar patches of different manifolds are similar. It is shown that Laplacian Eigenmap possesses both properties [37].

The Laplacian Eigenmap is based upon graph Laplacian and its algorithm consists of three major steps including (1) constructing an adjacency graph, (2) choosing the weights, and (3) finding Eigenmaps [50,57]. Each step involves choosing an appropriate way of implementation and determining a proper value for multiple parameters. Although the right choice of parameters to obtain the most accurate embedding depends on the application, existing options and the effect of choosing each one are discussed in the next section thoroughly. To conclude, the locality preservation and structural equivalence properties, which are guaranteed by Laplacian Eigenmap, are the key determinant factors to our proposed method.

## 3. Methods

The objective of multi-modal to mono-modal transformation presented in this article is to transform multi-modal input images into a similar intensity coordinate system. The general idea of this intensity transformation is to study the structure of small image patches by extracting more information than just the intensity of a single pixel. As the internal structures across images from different modalities are similar, we expect the transformation to result in a similar structural representation of multi-modal images.

**Figure 3:**
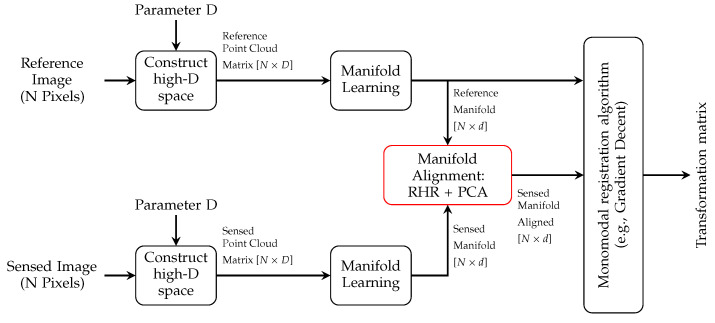
Pipeline of the proposed medical image registration using multi-modal to mono-modal transformation.

The proposed intensity transformation method consists of three main steps, i.e., constructing high dimensional space, studying the internal structure of an image using Laplacian Eigenmap, and manifold alignment. The main contribution of the proposed transformation can be summarized in the straightforward and efficient manifold alignment combined with improvements that we have made in the implementation of Laplacian Eigenmap for the purpose of image registration, even in cases where the sensed image only contains a portion of the object of interest. After the intensity transformation, a well-founded mono-modality image registration method finds the transformation matrix which, will align the input images. The general pipeline of the proposed method, presented in Figure 3, will be discussed in more detail in the following.

### 3.1. Constructing High-Dimensional Space

**Figure 4:**
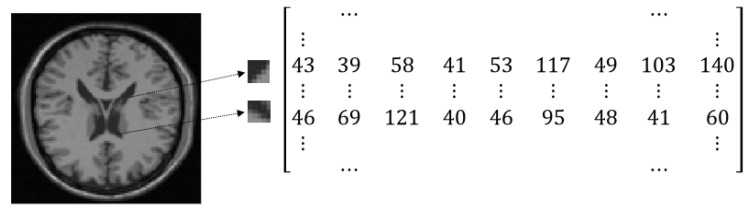
Matrix representation of a point cloud in high dimensional space; For an image of size 200×200 and patch size of 3×3, the matrix is 40,000 × 9.

In the problem of image registration only two images, reference and sensed, are present. A point cloud in high dimensional space is constructed by taking small patches from each input image. Each dimension of the high dimensional space is dedicated to one image pixel of every patch. Considering each patch of size s×s is moved one pixel from the previous patch, for an image of size $n_{r}\times n_{c}$, high dimensional manifold includes $n_{r}\times n_{c}=N$ points in an $s^{2}=D$ dimensional space. Such a set of points can be represented in matrix format using the coordinates of points. Figure 4 represents an example of constructing a high-dimensional point cloud using small patches of an image and the corresponding matrix representation of the point cloud. For a gray-scale image of size *N* pixels with patches of size *D* pixels, the matrix size is [N×D].

**Figure 5:**
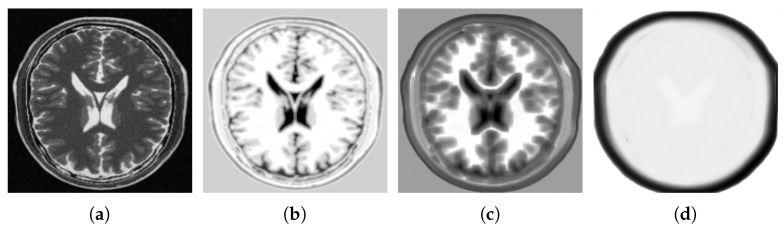
(**a**) A slice of T2-MRI image and it’s image representation of the embedded manifold with: (**b**) D=9; (**c**) D=49; and (**d**) D=225. The higher the *D*, the more blurred/general structural representation.

The choice of *D* depends on the application and size of the image. As we will see in the following, the embedded manifold resembles the original image when *D* is chosen relatively small. However, choosing a large value for *D* results in the blurred version of the image. In Figure 5a, a slice of a T2-MRI image is shown. The image has been studied by Laplacian Eigenmap with several *D* values. The effect of the selected value for parameter *D* on the sharpness of the rendered image after embedding the manifold is illustrated in Figure 5b–d. It is observed that as *D* increases the sharpness of the embedded manifold decreases. As can be seen from Figure 5d, using patch size 15×15 (as offered in [37]) leads to the loss of detail inside the brain structure.

### 3.2. Manifold Learning

The first step in dimensionality reduction with Laplacian Eigenmap requires constructing an *undirected adjacency graph* G=(V,E) with a set of points $x_{i}\in V$ in high dimensional space $R^{D}$ and a set *E* of edges that specifies neighborhood connectivities. In the following, terms point and node are used interchangeably when we are talking about high-dimensional space and graph, respectively. With a given set of points, there are two ways to determine neighborhood in the adjacency graph.

In the first approach, two points $x_{i}$ and $x_{j}$ are considered neighbors if they are in less than ϵ distance from each other. That can be formulated as $∥x_{i}-x_{j}{∥}^{2}<\epsilon$ where the norm is the usual Euclidean norm in $R^{D}$. This method, which is called *ϵ-neighborhood*, makes a symmetric graph; however, tends to construct a graph with several connected components. Besides, choosing the parameter ϵ can be challenging [50]. If ϵ is chosen relatively small, the graph will contain multiple connected components and will eventually affect the convergence of the eigenvalue problem in the last step. On the other hand, picking a relatively large value for ϵ affects the smoothness of the manifold as well as the sparsity of Laplacian matrix. Therefore, it demands a high memory/computational cost. Additionally, an advanced knowledge of the distance between all points is required.

Alternatively, the graph is constructed by finding *k*-nearest neighbors of each point in the high dimensional space and specifying an edge between the point and each of its’ neighbors [50]. This method, called *k-Nearest Neighbor* (parameter k∈N), tends to construct a directed graph, which is the case when node *i* belongs to the set of *k*-nearest neighbors of node *j* but, node *j* is not in *k*-nearest neighbors of node *i*. In the mathematical theory behind the Laplacian Eigenmap method, it is assumed that the graph is undirected/symmetric. Therefore, it is very important to force the graph to become symmetric. For that purpose, we employ two terminologies from the graph theory. We generate an undirected graph by either adding more edges wherever there is a one-way edge (called *symmetric kNN graph*) or deleting one-way edges (called *mutual kNN graph*) [58]. Either way, the number of neighbors of each point will change from the initial *k* value. In this approach, the graph is more likely integrated. Also, the choice of parameter *k* is less challenging as it has relatively less influence in both the convergence of eigenvalue problem and the accuracy of the ultimate result. The only concern about parameter *k* is that it affects the sparsity of Laplacian matrix. The parameter needs to be large enough to provide a graph with a single connected component and to preserve the general structure of the manifold, and not too large to demand too much memory and time to process.

In this article, for the purpose of constructing the adjacency graph, we use the combination of the *k*-nearest neighbor method with symmetric kNN graph. Mainly due to the convenient choice of parameter *k* and the tendency toward a single integrated graph. In short, two points *i* and *j* are neighbors if one of them is in *k*-nearest neighbors of the other one. The choice of parameter *k* is less critical in the process of dimensionality reduction. It simply is a trade off between memory/speed and assuring to have a single connected component. As medical scans use a smooth range of intensity levels which causes to obtain a smooth high-dimensional point cloud, tentatively, it is likely to obtain a single connected component with k=20 for medical scans of size around 200 × 200 pixels. In case of a noisy input image which may result in a graph of multiple connected components, employing a low-pass filter or increasing *k* is recommended.

The second step involves selecting the weight of each edge, which determines the power of the connectivity. In ref. [50], it is discussed that graph Laplacian is comparable to the Laplace-Beltrami (L-B) operator on manifolds. For the purpose of the best approximation of the L-B operator, it is recommended to use the following *heat kernel weighting scheme*:

(4) $$w_{ij}=\left\{\begin{array}{c} \begin{array}{cc} e^{\left(-\frac{{∥x_{i}-x_{j}∥}^{2}}{2\sigma^{2}}\right)} & ,\mathrm{if}\;(i,j)\in E\\ 0 & ,\mathrm{otherwise} \end{array} \end{array}\right.$$

where σ∈R is the only parameter. In the simplest case, so-called *simple minded weighting scheme*, σ=∞ and therefore, $w_{ij}=1$ if two nodes *i* and *j* are connected with an edge, and $w_{ij}=0$ otherwise.

The distance between neighboring points is a good measure of local information. Therefore, it is more desirable to keep the effect of anisotropic exponential weighting scheme by choosing a proper value for parameter σ. In the exponential weighting scheme of Equation (4), nearby points have a higher weight than distant points. Selecting a value for parameter σ is fairly challenging and data-dependent [59]. When σ is relatively large compared to $∥x_{i}-x_{j}∥$ then the weight of all connections is close to one which results in unweighted adjacency graph. On the contrary, a relatively small σ leads to non-significant edges which may later cause failure in the convergence of the eigenvalue problem. The right choice for σ lies in between these two extremes [59].

In ref. [60], it is proposed to construct the σ-dependent weight matrix $W(\sigma^{2})$ and compute

(5) $$S\left(\sigma^{2}\right)=\sum_{i=1}^{n}\sum_{j=1}^{n}w_{ij}\left(\sigma^{2}\right)$$

for several values of σ. Then plot *S*-curve versus $\left(\sigma^{2}\right)$ in log-log scale. The *S*-curve has two asymptotes at σ=0 and σ=∞ and has been used to choose σ where the graph appears linear in the upper half of the total range.

The idea proposed in [60] and Equation (5), adds extra processing time for each image to compute the S-curve. We propose to choose the variance of the heat kernel equal to the maximum squared distance of all edges. In other words:

(6) $$\sigma^{2}=\max_{allx_{i},x_{j}\in V}(∥x_{i}-x_{j}{∥}^{2}).$$

In this case, all edge weights are bounded between $e^{{}^{-1}{/}_{2}}\approx 0.606$ and 1. While reducing the computing time, empirically, the embedded manifolds and the registration outcome are valid and reliable.

In ref. [50], the optimal embedding attempts to map points $x_{i}$ to points $y_{i}$ while preserving local information. In other words, connected points $x_{i}$ and $x_{j}$ will stay as close together as possible after being mapped to $y_{i}$ and $y_{j}$, respectively. This statement is formulated as minimizing the following objective function:

(7) $$\sum_{ij}{\left(y_{i}-y_{j}\right)}^{2}w_{ij}.$$

With a given symmetric *weight matrix* W with entries $w_{ij}$, we define the diagonal *degree matrix*D such that $d_{ii}=\sum_{j}w_{ij}$. The entries of the degree matrix D are column (or row, since W is symmetric) sums of W. We also define the *Laplacian matrix* as L=D−W. If the set *V* contains *N* points, then W and therefore, D and L are all sparse N×N symmetric matrices.

By adding the orthogonality constraint $y^{T}D1=0$ in order to eliminate the trivial solution and the constraint $y^{T}\mathrm{Dy}=1$ for removing an arbitrary scaling factor in the embedding, the minimization problem of Equation (7) simplifies to:

(8) $$\underset{\begin{array}{c} y^{T}\mathrm{Dy}=1\\ y^{T}D1=0 \end{array}}{\arg\min}y^{T}\mathrm{Ly}.$$

The solution vector y to Equation (8) is obtained by the minimum eigenvalue solution to the generalized eigenvalue problem [50]:

(9) Ly=λDy.

The Equation (9) is called *eigen-decomposition* or *spectral decomposition* of L-B operator, whereby the matrix is represented in terms of its eigenvalues and eigenvectors. The set of eigenvalues is called the *spectrum* of L [61]. The most important property of the eigenvalues and eigenvectors of L-B operator is that they are *isometric invariant*. In other words, if the manifold is not stretched (e.g., bent into extra dimension), the spectral values will not change. Hence, two manifolds with different orientation will have similar spectral representations, provided the underlying topology of two mesh graphs [62].

Equations (4) and (7) make it clear that L is real symmetric and positive semi-definite. All eigenvalues of a real symmetric matrix are real and eigenvectors corresponding to distinct eigenvalues are orthogonal. As L is positive semi-definite, all eigenvalues are non-negative ($\lambda_{i}\geq 0$ for all *i*) [63]. Every row sum and column sum of L is zero. In consequence, eigenfunction **1** and eigenvalue λ=0 are trivial solutions to Equation (9). The multiplicity of eigenvalue zero is associated with the number of *connected components* of the graph [57]. For a graph with multiple connected components, L is a block diagonal matrix, where each block represents the Laplacian matrix of a connected component, possibly after reordering the vertices [64].

In the last step, having the properties of the Laplacian matrix mentioned earlier in mind, we need to leave out all eigenvectors corresponding to eigenvalues equal to zero and use the next *d* eigenvectors with the smallest non-zero eigenvalues for embedding the manifold in a low *d*-dimensional Euclidean space [50]. The embedded manifold will be in the form of an N×d matrix $Y=[y_{1},y_{2},\ldots ,y_{d}]$ where the *i*th row demonstrates embedding coordinates of the *i*th point.

There are many different algorithms available for finding *d*-smallest eigenvalues of the generalized eigenvalue problem. Among them are the Householder method [65,66], the Q.R. method [66], subspace iteration [67,68], etc. Many of these algorithms are inefficient when applied to very large structural systems [69]. The most efficient algorithms are implemented in ARPACK software. ARPACK, which stands for ARnoldi PACKage, is very powerful in approximating a few eigenvalues and corresponding eigenvectors. As the matrix L is symmetric, the Arnoldi process is reduced to a variant of the Lanczos process called the Implicitly Restarted Lanczos Method (IRLM) [70]. Computational approximations performed by eigen-solvers makes the smallest eigenvalue not to be exactly equal to zero. However, finding the number of connected components from the Laplacian matrix is straightforward and leads us to discard as many eigenvectors as needed. The first *d*-eigenvectors, corresponding to the smallest eigenvalues greater than zero are orthogonal bases for embedding manifold in lower dimension space. At this point, *d* eigenvectors of size N×1 locate *N* points of the embedded manifold in an *d*-dimensional space.

**Figure 6:**
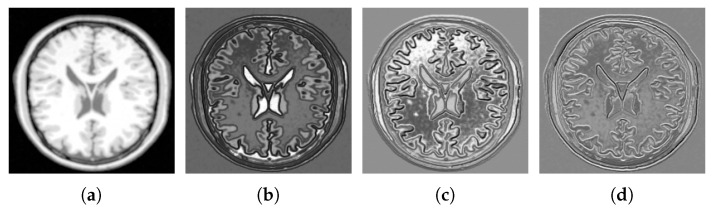
Structural representation of T1-MRI using Laplacian Eigenmap. Feature images indexed 1, 6, 11, and 18 are shown in (**a**–**d**), respectively.

A structural representation of an input image is obtained by reordering an eigenvector into the size of the original input image. Each eigenvector represents input image with a specific degree of derivation. The index of the eigenvector increases, structural representation represents a higher degree of the derivative. As structural images are associated to distinct eigenvalues from the spectrum of the image manifold, from now on, they are shortly referred as *feature images* [71]. Structural representations of a human brain T1-MRI using feature images 1, 6, 11, and 18 are shown in Figure 6. The necessary steps toward dimensionality reduction using Laplacian Eigenmap are summarized in Algorithm 1.

**Algorithm 1:** Summary of dimensionality reduction (structural representation) with Laplacian Eigenmap. **Input**: A set of *N* points $X={\left(x_{1},\ldots ,x_{N}\right)}^{T}\in R^{N\times D}$ in high-dimensional space **Output**: A set of *N* points $Y={\left(y_{1},\ldots ,y_{N}\right)}^{T}\in R^{N\times d}$ in low-dimensional space **1** Compute the distance between every two data points; **2** Construct the adjacency graph by considering *k*-nearest neighbor with the choice of *k* and symmetric kNN graph; **3** Define bandwidth $\sigma^{2}=\max(∥x_{i}-x_{j}{∥}^{2})$ for $\forall x_{i},x_{j}\in V$; **4** Assign weight to each edge between every two neighbors using heat kernel weighting scheme; **5** Construct the sparse, real, and symmetric N×N matrices W, D, and L; **6** Find the number of connected components (nConComp) from L; **7** Solve the generalized eigenvalue problem Ly=λDy for (nConComp+d) eigenvalues and the corresponding eigenvectors; **8** Sort eigenvectors to represent the eigenvalues in increasing order; **9** Leave out the first nConComp eigenvectors; **10**
**Return** the remaining *d*-eigenvectors;

### 3.3. Manifold Alignment

The Lanczos algorithm starts with a random initial vector. Therefore, the embedding in low dimensional space is arbitrary. In other words, although Laplacian Eigenmap preserves local information, it does not guarantee alignment of embedded manifolds in the low dimensional space. Consequently, a manifold alignment step is required [38]. Several manifold alignment techniques have been introduced [72,73,74,75]; however, a linear alignment technique, e.g., PCA, is pertinent in this application.

**Figure 7:**
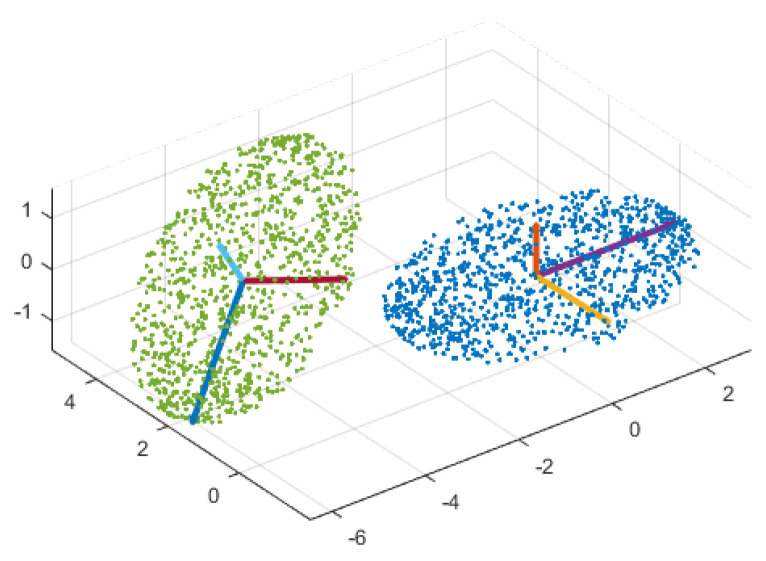
Right-hand rule inspection of two embedded manifolds. The first three principal directions of the reference (blue) and the sensed manifolds (green) follow the left-hand and the right-hand rule, respectively.

PCA provides parameters of a rigid-body transformation consisting of translation and rotation. Since the negative of any eigenvector is a valid eigenvector, the mapping between two embedded manifolds may require a flip. Rigid-body transformation using PCA is not capable of dealing with such random sign ambiguity. If principal components of both manifolds follow the same either right-hand or left-hand rule defined between perpendicular principal vectors using the cross product of vectors, PCA will align them with adequate precision. Such a sign ambiguity is shown in Figure 7.

We propose a fast and straightforward way to examine whether both reference and sensed embedded manifolds comply with the right-hand rule. The significance of the proposed criterion is that it is also applicable in any *d*-dimensional space. The sign of the covariance associated with every two corresponding feature image is a measure of the direction of their association. If they have negative association, we multiply (−1) with the feature image of the sensed embedded manifold.

When the reflection error is compensated with the proposed idea, PCA computes rigid-body transformation matrix **R** which aligns centered embedded manifolds as follows:

(10) RA=B

where, matrices A and B are the principal directions of sensed and reference manifolds, respectively. As the matrix A is orthonormal, according to properties of PCA, the rotation matrix simplifies to:

(11) $$R=BA^{-1}=BA^{T}\;.$$

In summary, the alignment is obtained by moving the center of both manifolds to the origin, followed by rotating principal directions of one manifold (*sensed*) to align with principal directions of the other manifold (*reference*) [56]. Rigid-body transformation of sensed embedded manifold to align with reference embedded manifold (after examining right-hand rule compliance) using PCA can be formulated as:

(12) $${p_{s_{i}}}^{\prime }=R\left(p_{s_{i}}-c_{s}\right)+c_{r}$$

where $c_{s}$ and $c_{r}$ are centroids of sensed points $p_{s_{i}}$ and reference points, respectively. The outcome of manifold alignment is two series of feature images which use comparable relative intensity levels to generate the necessary contrast in an image.

**Figure 8:**
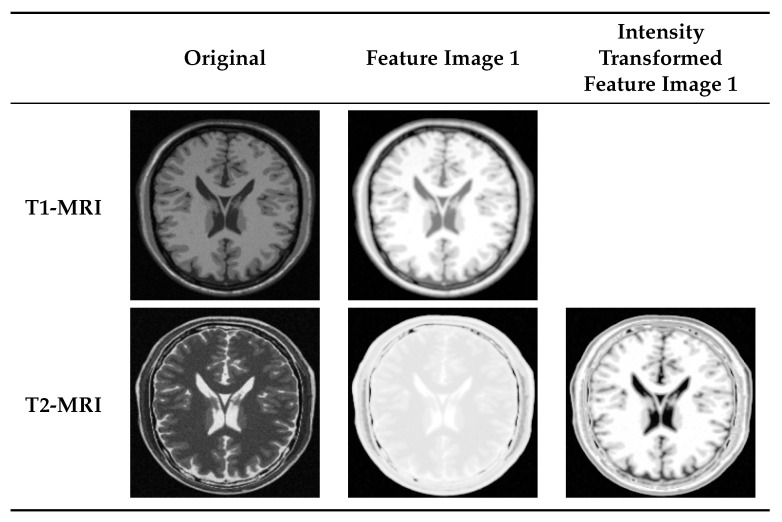
Transforming multi-modal scans into a same intensity coordinate system. Even though original T1- and T2-MRI have intensity variations, T2- intensity transformed feature image uses comparable relative intensity levels as T1-MRI feature image to generate the necessary contrast in the image.

Figure 8 shows the necessity of manifold alignment to complete the expected intensity transformation. Different modalities use different relative intensity levels to display a same part of the brain. Please note the difference in how the CSF, white matter, and gray matter are displayed in T1- and T2-MRI images. The necessary intensity adjustment is not yet achieved after manifold learning. However, manifold learning followed by manifold alignment performs an intensity transformation on both images such that they use comparable intensity levels (please note the color similarity between T1-MRI feature image 1 with T2-MRI intensity transformed feature image 1 in Figure 8).

### 3.4. Registration

The multi-modal to mono-modal transformation using manifold learning followed by manifold alignment provides the structural representation of multi-modal scans. The new representation, instead of the original raw images, is applicable in finding the registration parameters using mono-modal registration techniques. Later, the estimated transformation matrix is used to align the original multi-modal scans.

In this work, we use an intensity-based and a Fourier–Mellin based [21] image registration for the problem of image registration with full and partial data, respectively. Intensity-based registration utilizes regular step gradient descent optimizer iteratively in order to adjust the transformation parameters so that the optimization follows the gradient of mean squares in the direction of the extrema.

## 4. Experimental Validation

In this section, we evaluate the performance of our proposed intensity transformation method using Laplacian Eigenmap and the proposed manifold alignment technique in improving and facilitating the multi-modal medical scans registration, in situations either full or partial data is available. To do so, we designed and carried out three sets of experiments.

### 4.1. Experimental Setup

The present experimental studies were implemented using MATLAB R2013a environment running on a personal computer with Intel(R) Xeon(R) E3-1245 CPU @ 3.50 GHz and 32 GB memory. To validate the effectiveness of the proposed idea, we exploited two standard, widely used and publicly available datasets of simulated and real human brain scans. We also utilized elastix toolbox [76,77] for NMI-based registration. In our experiments, NMI as well as Mattes MI metrics were computed using all pixels with 50 histogram bins.

We performed experiments on T1-, T2-, and PD-weighted MR images from the BrainWeb database (http://www.bic.mni.mcgill.ca/brainweb/) [78,79,80,81]. The images in our experiments contained 3% noise and 20% intensity non-uniformity. We also made use of the CT, and different MR modalities from the Vanderbilt Database which was collected in Retrospective Image Registration Evaluation (RIRE) project (http://www.insight-journal.org/rire/index.php) [82,83,84]. Moreover, RIRE database provides rectified images (rectified) that were generated by using [85] for correcting the static field inhomogeneity. In the current work, all images were resized to 200×200 to be cost-effective in terms of processing time and memory usage.

### 4.2. Multi-Modal to Mono-Modal Transformation

In the first set of experiments, we aim to visually evaluate the performance of the proposed intensity transformation in which, similar relative intensity levels as in the reference feature image will be used to redraw the sensed feature image. We employed images from different datasets to inspect the effectiveness of the method aside from patient-dependent similarities in images.

**Figure 9:**
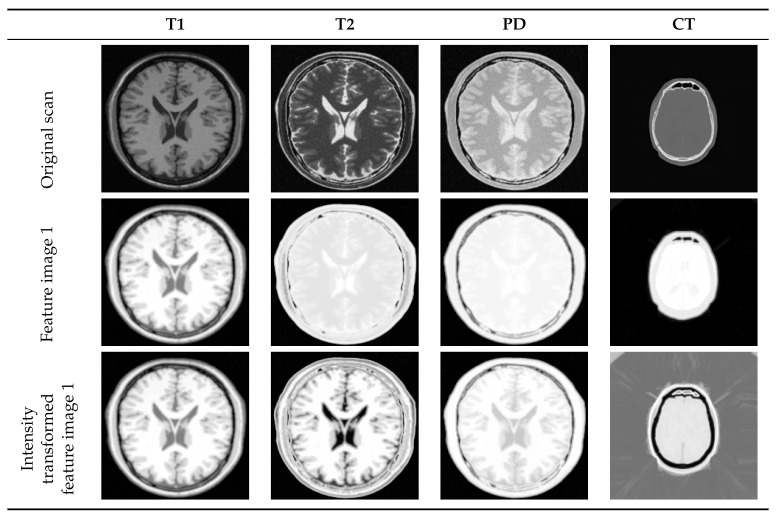
Intensity transformation of multi-modal medical scans. (**Top row**) shows the original MRI and CT scans of the human brain; (**Middle row**) shows the first feature image of each modality after manifold learning; (**Bottom row**) shows the same feature images after manifold alignment. Intensity mapping of feature images obtained from T2-, PD-MRI, and CT are matched with the one obtained from T1-MRI.

We studied the manifold of each image independently. First, we used patches of size 3×3, resulting in a manifold containing 40,000 points in a 9-dimensional space. Then, we constructed the Laplacian graph with k=20 nearest neighbors of each point and embedded the manifold in a three-dimensional space. Manifold alignment was performed by keeping the T1-weighted MRI as the reference and aligning the rest along with it. Figure 9 represents the result of multi-modal to mono-modal transformation. From the bottom row, it is clear that after manifold alignment, all modalities are using similar intensity mapping as of T1-MRI-feature image 1, as expected. It is useful to note that a similar intensity transformation is possible with the user’s choice of reference image.

### 4.3. Multi-Modal Registration with Full Data

To assess the efficacy of the proposed method in registering multi-modal images with full data, two sets of experiments were carried out exploiting RIRE dataset. In both studies, as the proposed method with the current implementation is only feasible for 2D images, slice-wise alignment is obtained. First, the high-dimensional manifold of each image was studied separately using the same set of parameters including D=9, k=10, and d=3. Then, the proposed manifold alignment technique was conducted to transform multi-modal scans into a mono-modal intensity coordinate system. Next, rigid-body registration parameters were estimated using the first feature image of each modality. To do so, we utilized regular step gradient descent optimizer along with mean square error metric as a method of mono-modal registration. In the end, we employed the registration parameters on the original set of images.

**Figure 10:**
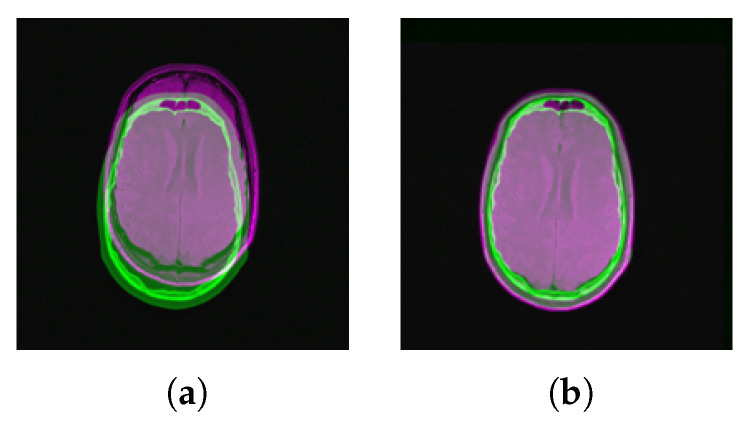
Pairwise display of a sample CT and PD-MRI scans from RIRE database. (**a**) Unregistered (MI = 0.8276), (**b**) Registered using the proposed transformation followed by mono-modal registration (MI = 1.1715).

In the first set of experiments, we examined the performance of registering CT scans with multiple MR modalities using the proposed method. As the ground truth information for the RIRE testing dataset was not available, we measured the similarity metric MI between image pairs before and after registration. MI quantifies the degree of statistical dependency between the reference and the sensed images. The current dataset contains CT, T1-, T2-, and PD-MRI scans of 10 patients. Only 6 of them have the rectified version of MRIs. Table 1 reports mean and standard deviation of MI measured before and after registration for each pair of the CT-MR scan. The results are statistically significant with (p<0.5%). Figure 10 presents an example pairwise display of CT and PD-MRI scan before and after registration with their MI values. It demonstrates the improvement of MI value resulting from the proposed method, even without the MI value being computed and optimized during registration.

To further evaluate the competence of our method quantitatively, we designed the second set of experiments. We utilized RIRE training dataset, as it contains the ground truth aligning information. We created a non-aligned slice of CT (sensed image) using randomly generated rotation angle in the range of [−π/4,+π/4] and translation along each axis, constrained to keeping the brain region in the frame. Then, the distorted image was registered using three different methods: (1) the proposed method in [37,38] using Laplacian images; (2) multi-modal registration with Mattes MI metric [86] and One plus One Evolutionary [87] optimizer; (3) the proposed method. Then, the registration error was computed using the Mean Absolute Error (MAE) of 5 random relocated points. Since RIRE training dataset has multi-modal images of only one patient, we repeated the experiment 30 times for each pair of modalities. Table 2 reports mean and standard deviation of the distance error (in millimeter). Bold-faced numbers show the best result obtained for each modality pair. All the results presented except those with asterisks are statistically significant with (p<0.1%). Registration error of CT-T1 rectified scans is statistically significant with (p<0.3%). Registration error of CT-T2 rectified scans using proposed method is higher than using MI; nonetheless, it is not statistically significant.

### 4.4. Multi-Modal Registration with Partial Data

We assessed the role of intensity transformation in the success of partial image registration. In the problem of co-registration of multi-modal medical scans with partial data, while images are acquired from different modalities, the sensed (partial data) only covers part of the reference (full data) and we seek to locate the small one within the space of the larger one.

The experiments were carried out as follows. First, synthetic examples of partial data were generated by rotating, translating, and cropping the sensed image with random parameters. For some experiments, extra scaling was added to the sensed image. Next, both images were studied with the proposed technique (Figure 3) and then a feature image of each was passed on to the FMT algorithm. Once they are available, the FMT algorithm computes the transformation parameters (translation, rotation, and scale) accordingly. This step requires fine-tuning the FMT algorithm. Finally, the co-registration was performed on the original full and partial sensed images using the estimated transformation parameters.

**Figure 11:**
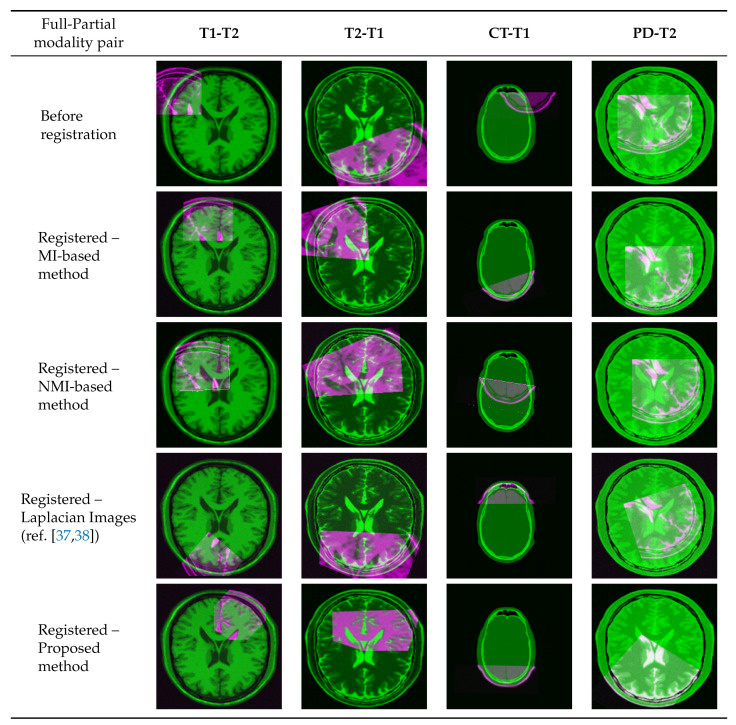
Pairwise display of multi-modal image registration with partial data.

The performance of the registration is investigated qualitatively with a pairwise display of original and registered images in Figure 11. It’s worth mentioning that none of the following results were feasible by using FMT without benefiting from manifold learning and multi-modal to mono-modal transformation. Hence, comparison of registration error is not reported.

**Figure 12:**
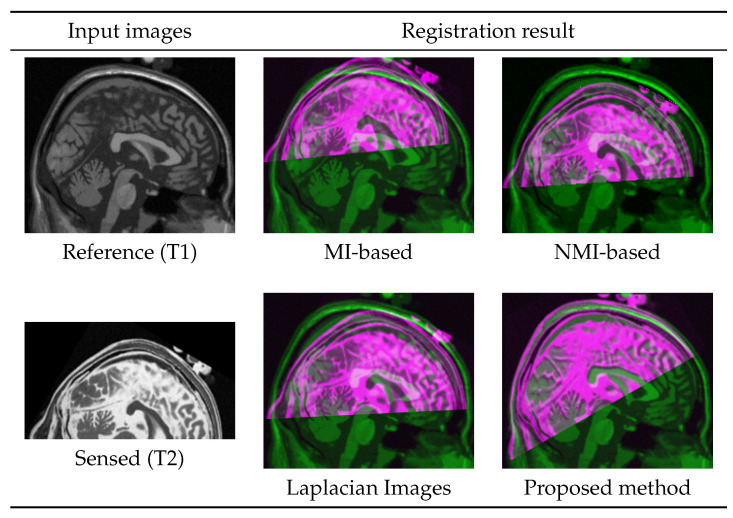
Registration of a synthetic example with partial data in the sensed image.

In a more clinically related scenario, a T1-MRI of a patient from a past scan (reference) is registered with a newly captured T2-MRI (sensed) that has covered part of the patient’s brain. Both images were acquired in the axial direction; however, they are reviewed and registered in the sagittal direction. While taking the new scan (T2), the patient was positioned differently from T1 on the imaging bed. The reference and sensed images, as well as registration result from multiple methods, are presented in Figure 12. Images do not represent a real patient and are synthetically generated using the BrainWeb database.

## 5. Discussion and Conclusions

Medical image registration has been a very longstanding research over the past 15 years, and it has hugely contributed to our understanding and interpretation of medical images. Its’ application broadly lies in a variety of medium and high-level image analysis tasks, such as segmentation, object localization, panoramic image creation, object detection, and diagnostics analysis of X-ray, MRI, and CT images. While computational image registration methods have been widely available in the medical image analysis community, it is almost impossible to design and develop a general registration method which is optimized for all use cases. The present work discusses a large body of recent progress in medical image registration with a great emphasis on utilizing different computational algorithms to develop a multi-modal to mono-modal transformation technique, providing a significant capability in the multi-modal registration of medical images. Advancement in the proposed method is applicable for images with either full or partial overlap.

There are a number of advantages to the current contribution that we discuss here. The presented multi-modal to mono-modal transformation can be used as a general preprocessing step, regardless of the overlapping of images. Moreover, it facilitates recovering strong scales, rotations, and translations. In the context of medical image registration strategies, the presented method is considered as a parametric one, however, configuring and assigning the exact value to the parameters is not essential. On the other hand, this gives some degree of freedom for further tuning the smoothness of the high-dimensional manifold, which will eventually affect the convergence rate of the eigenvalue problem positively. In support of accurate structural representation, with the use of the proposed multi-modal to mono-modal transformation, multiple feature images (maps) are generated as the output of the structural representation from which we can select. Although we evaluated the performance of the proposed transformation by only considering the rigid transformation of human brain images (except registration with partial data that considered affine transformation), we believe that registering images of other organs as well as non-rigid registration can benefit from the same concept.

Two computational bottlenecks of the proposed method are: (a) the construction of the adjacency graph; (b) solving the eigenvalue problem. In our experiments and with the system specification mentioned in Section 4.1 (high performance desktop with 32GB memory), for an image size 200×200 with k=10, the average time to construct the adjacency graph was 1, 12, and 114 s with D=9, 25, and 225, respectively. The computational time to find the eigenvectors depends significantly on the smoothness of the manifold and the sparsity of the Laplacian matrix (parameter *k*). On average, for k=10 to 500, it can vary from 15 to over 300 s. It is good to note that increasing the number of dimension from 2D to 3D leads to a significant increase in the size of the Laplacian and degree matrices. For example, for a 3D scan with only $n_{s}=30$ slices, the size of the L and D will be 900 times bigger. Even though the time complexity of finding a pairwise distance between *N* points is $O(N^{2})$ (depending on the implementation algorithm), the convergence rate of Eigen-solvers limits the extension of this method to 3D space.

There is also a list of limitations to this study. First of all, as explained earlier, the current implementation is computationally expensive, which makes it inefficient for 3D volume registration. Second, the proposed pipeline requires extra processing time for learning the structure of the input images. The solutions to these limitations are beyond the scope of this paper and require more advanced computational methods (e.g., super-pixels) as well as efficient computational platforms (e.g., parallel processing on top of high-performance clusters). Furthermore, since the inner structure must be present in both modalities, the proposed transformation applies to images of anatomical modalities.

At the expense of time for computing structural representation, we obtained higher accuracy in rigid-body registration of multi-modal images with full data. More importantly, a new door is opened to the registration of partially overlapped multi-modal images. In the future, we plan to investigate the effectiveness of the proposed intensity transformation in registering multi-modal partially overlapped images quantitatively. In the current implementation, we took advantage of Laplacian Eigenmap as a method of nonlinear dimensionality reduction. However, preliminary experiments indicate some other methods of manifold learning, e.g., Isomap and LLE, are also capable of intensity transformation of multi-modal images. 

## Figures and Tables

**Table 1:** Mean and standard deviation of mutual information computed for each pair of the CT-MR scan before and after registration using the proposed idea. Details can be found in the main text.

Modality Pair	Before Registration	After Registration
CT-PD	0.8752 ± 0.15	1.2084 ± 0.22
CT-T1	0.8685 ± 0.15	1.1863 ± 0.23
CT-T2	0.8151 ± 0.10	1.0800 ± 0.20
CT-PD Rectified	0.7846 ± 0.08	1.1132 ± 0.09
CT-T1 Rectified	0.7759 ± 0.06	1.0958 ± 0.08
CT-T2 Rectified	0.7736 ± 0.06	1.0450 ± 0.06

**Table 2:** Mean and standard deviation of distance error (in millimeter) for each pair of CT-MR modality scans. Images are aligned using Laplacian images [37,38], MI-based registration, and the proposed transformation followed by mono-modal registration.

Modality Pair	Laplacian Images (ref. [37,38])	MI-Based Reg. w/o Intensity Trans.	Mono-Modal Reg. w/ Proposed Trans.
CT-PD	3.5622 ± 0.05	2.0239 ± 0.14	**1.3740 ± 0.09**
CT-T1	2.9912 ± 0.11	1.3873 ± 0.18	**1.0042 ± 0.11**
CT-T2	1.9467 ± 0.15	1.7335 ± 0.12	**1.4137 ± 0.09**
CT-PD Rectified	2.3318 ± 0.12	1.3260 ± 0.21	**1.1462 ± 0.11**
CT-T1 Rectified	2.0075 ± 0.07	0.9727 ± 0.39	**0.7424 ± 0.22** *
CT-T2 Rectified	1.9447 ± 0.33	**0.9294 ± 0.23**	0.9922 ± 0.16 **
